# Effects of Acute and Sub-chronic Exposure to Low Doses of Methyl-tertiary Butyl Ether on mRNA Levels of Three Members of Glutathione S-transferases in Liver and Testis of the Male Rats

**Published:** 2018-06

**Authors:** Ahmad Ali BADR, Mostafa SAADAT

**Affiliations:** Dept. of Biology, College of Sciences, Shiraz University, Shiraz, Iran

## Dear Editor-in-Chief

Methyl-tertiary-butyl ether (MTBE) is used to reach clean air standards. Exposure to MTBE is associated with several adverse effects on human health. Enzymes of phase II metabolism, such as glutathione S-transferase superfamily (GST, EC 2.5.1.18) may modulate the biological effects of the ROS. The expression levels of the GSTs members may significantly alter in cells exposed to some drugs (1, 2) or electromagnetic fields (3). There are a few studies with inconsistent results, investigating the effects of MTBE on enzyme activity or mRNA levels of phase II metabolism enzymes, including the members of GST super-family (4–6). The effect of high doses of MTBE on mRNA levels of glutathione S-transferases (*Gstm1, Gstt1* and *Gstp1*) was reported (5, 6). In general populations, people usually exposed to low or very low levels of MTBE (7). The present experiments were carried out to determine the effect(s) of acute and sub-chronic exposure to low doses of MTBE on mRNA levels of *Gstm1, Gstt1* and *Gstp1*.

This study was carried out in accordance with the Code of Ethics of the World Medical Association (Declaration of Helsinki) for experiments involving animal experiments and it is approved by Ethics Committee of Shiraz University.

For acute exposure, male Wistar rats (180–200 gr) were randomly divided into 3 equal sub-groups (n=5) and received a single dose of MTBE (0, 10 and 30 mg MTBE/Kg). In sub-chronic exposure, rats were randomly divided into 2 equal subgroups (n=5) and received 0 and 10 mg MTBE/Kg/day for 28 consecutive days. Animals were housed in plastic cages under standard animal room conditions with a 12 h light/dark cycle and a temperature of 25±2 °C. They received standard pellet food, and tap water was available *ad libitum*. Details of RNA extraction and real-time PCR conditions were reported in our previous report (5).

**Fig. 1:**
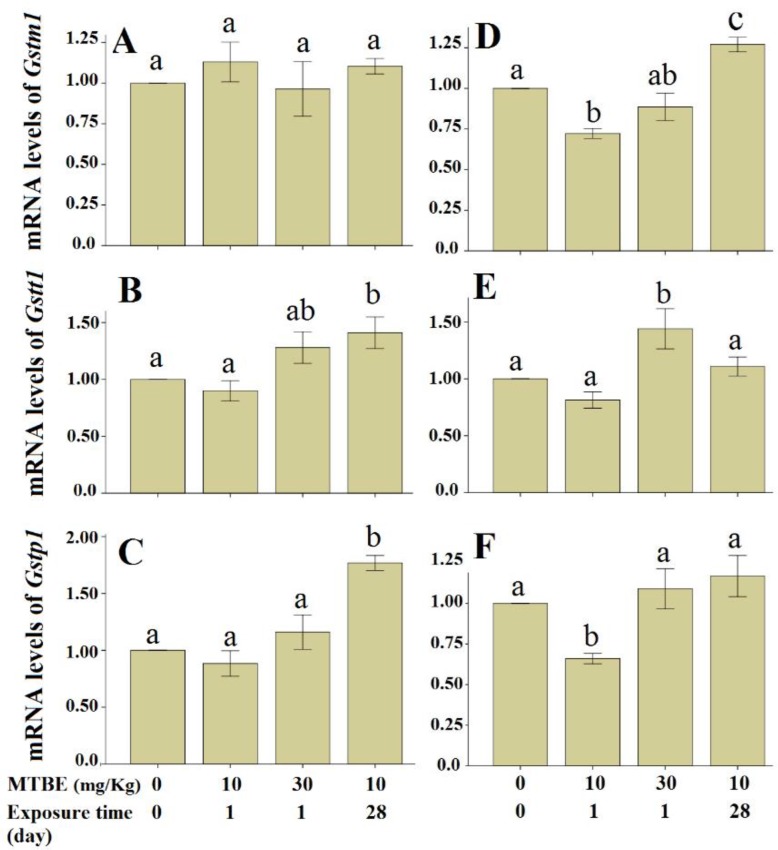
Relative expression levels of *Gstm1*, Gstt1 and *Gstp1* in liver (A–C) and testis (D–F) of rats exposed to various doses of Methyl-tertiary-butyl ether (MTBE) (*n*=5, mean ± *SE*). All values of mRNA levels were compared with control group (=1). Data were analyzed by one-way analysis of variance (ANOVA) followed by Bonferroni post hoc test. Same alphabets mean no significant differences between experimental groups

Statistical analysis revealed significant differences in the mRNA levels of *Gstt1* (F=4.82; df=3, 16; *P*=0.014) and *Gstp1* (F=15.53; df=3, 16; *P*<0.001) between study groups. Bonferroni post hoc test revealed that the *Gstt1* and *Gstp1* mRNA levels were significantly increased in liver of rats sub-chronically exposed to 10 mg MTBE/Kg/day compared to the control group (Fig. 1 A–C).

The means of the *Gstm1* (F=21.49; df=3, 16; *P*<0.001), *Gstt1* (F=6.37; df=3, 16; *P*=0.005) and *Gstp1* (F=6.21; df=3, 16; *P*=0.005) showed significant differences between study groups. The mRNA levels of *Gstm1* and *Gstp1* significantly decreased in testis when rats were exposed to a single dose of 10 mg MTBE/Kg, whereas the mRNA levels of *Gstm1* significantly increased in testis of the experimental animals sub-chronically exposed to 10 mg MTBE/Kg/day. The *Gstt1* mRNA levels increased in testis of the rats exposed to a single dose of 30 mg MTBE/Kg (Fig. 1 D–F).

The mRNA levels of three members of GSTs (*Gstm1*, *Gstt1*, and *Gstp1*) did not show significant alteration under both acute (6) and sub-chronic exposure times (5). In the general population, people usually exposed to low or very low levels of MTBE (7), in the present study we used two low levels of MTBE. We found significant alterations in the mRNA levels of the examined genes (Fig. 1). Taken together, the alteration patterns of the mRNA levels of the examined GSTs are not similar when rats treated by low, moderate and high concentrations of MTBE.

The Kolmogorov-Smirnov test showed that the total amounts of MTBE used (TAMU= calculated by multiplying of MTBE dose and exposure time) had no normal distribution (Z=1.84, *P*=0.002), the Spearman’s rho correlation coefficients between TAMU and the mRNA levels were used. In liver, the mRNA levels of *Gstt1* (r=0.680, df=18, *P*=0.001) and *Gstp1* (r=0.668, df=18, *P*=0.001) and in testis, the mRNA levels of *Gstm1* (r=0.507 df=18, *P*=0.022) and *Gstt1* (r=0.493, df=18, *P*=0.027) showed positive correlation with the TAMU.

Although we found some statistical significant differences between MTBE exposed groups and control groups, the mentioned alterations are small (less than two-fold). Other studies should be carried out in order to find the biological significance of the small deviation from normal control levels.
